# Allergic rhinitis and its associated co-morbidities at Bugando Medical Centre in Northwestern Tanzania; A prospective review of 190 cases

**DOI:** 10.1186/1472-6815-12-13

**Published:** 2012-11-08

**Authors:** Said A Said, Mabula D Mchembe, Phillipo L Chalya, Peter Rambau, Japhet M Gilyoma

**Affiliations:** 1 Department of Surgery, Catholic University of Health and Allied Sciences Bugando, Mwanza, Tanzania; 2 Department of Surgery, Muhimbili University of Health and Allied Sciences, Dar Es Salaam, Tanzania; 3 Department of Pathology, Catholic University of Health and Allied Sciences Bugando, Mwanza, Tanzania

**Keywords:** Allergic rhinitis, Co-morbidities, Treatment outcome, Tanzania

## Abstract

**Background:**

Allergic rhinitis is one of the commonest atopic diseases which contribute to significant morbidity world wide while its epidemiology in Tanzania remains sparse. There was paucity of information regarding allergic rhinitis in our setting; therefore it was important to conduct this study to describe our experience on allergic rhinitis, associated co-morbidities and treatment outcome in patients attending Bugando Medical Centre.

**Methods:**

This was descriptive cross-sectional study involving all patients with a clinical diagnosis of allergic rhinitis at Bugando Medical Centre over a three-month period between June 2011 and August 2011. Data was collected using a pre-tested coded questionnaire and analyzed using SPSS statistical computer software version 17.0.

**Results:**

A total of 190 patients were studied giving the prevalence of allergic rhinitis 14.7%. The median age of the patients was 8.5 years. The male to female ratio was 1:1. Adenoid hypertrophy, tonsillitis, hypertrophy of inferior turbinate, nasal polyps, otitis media and sinusitis were the most common co-morbidities affecting 92.6% of cases and were the major reason for attending hospital services. Sleep disturbance was common in children with adenoids hypertrophy (*χ*^2^ = 28.691, P = 0.000). Allergic conjunctivitis was found in 51.9%. The most common identified triggers were dust, strong perfume odors and cold weather (P < 0.05). Strong perfume odors affect female than males (*χ*^2^ = 4.583, P = 0.032). In this study family history of allergic rhinitis was not a significant risk factor (P =0.423). The majority of patients (68.8%) were treated surgically for allergic rhinitis co morbidities. Post operative complication and mortality rates were 2.9% and 1.6% respectively. The overall median duration of hospital stay of in-patients was 3 days (2 – 28 days). Most patients (98.4%) had satisfactory results at discharge.

**Conclusion:**

The study shows that allergic rhinitis is common in our settings representing 14.7% of all otorhinolaryngology and commonly affecting children and adolescent. Sufferers seek medical services due to co-morbidities of which combination of surgical and medical treatment was needed. High index of suspicions in diagnosing allergic rhinitis and early treatment is recommended.

## Background

Allergic rhinitis is recognized as one of the most common otorhinolaryngological condition which has considerable effects on quality of life and can have significant consequences if left untreated [1]. Globally allergic rhinitis constitutes a worldwide public health problem with a prevalence of 10% to 40% and the trend is increasing [2-5]. About 20 to 40 million people in the United States alone are estimated to be affected, [6] with 3.5 million lost workdays and 2 million lost schooldays annually [1]. Local population-based studies have reported a prevalence of allergic rhinitis of 44% in Singaporean school children [7]. In UK intermittent allergic rhinitis (also known as seasonal allergic rhinitis or hay fever affects up to 30% of adults and 40% of children at some time in their lives [8].

Allergic rhinitis is associated with significant co-morbidities and health care costs [9] and has been identified as one of the top ten reasons for visits to primary care clinics [10]. The total burden of allergic rhinitis lies on impaired physical and social functioning and also in a financial burden for treatment of its co-morbid diseases [1]. Allergic rhinitis commonly causes sleep disturbance, fatigue, listlessness irritability, and poor concentration leading to developmental delay, impaired learning ability and poor school performance in children [11-14]. Socially unacceptable behavior such as sniffing, sneezing, noisy breathing and coughing may lead to isolation and rejection at school and home. Further morbidity results from associated conditions such as sinusitis, adenoids, tonsil hypertrophy and otitis media [11].

Time trend studies of the prevalence of allergies in Africa show a consistent increase over a period of 7–10 years [15]. In South African epidemiological data are scarce but the overall prevalence of allergic rhinitis in children is at least 20% to 24% and there is evidence of increasing prevalence [11]. In Tanzania the data concerning allergic rhinitis are limited. Observation from Bugando Medical centre E.N.T clinical records reveals that allergic rhinitis and its associated co morbidities are common problem encountered with increasing trend for the past three years. Despite adverse sequalae, allergic rhinitis remains unrecognized and miss managed condition. Further more many patients do not recognize allergic rhinitis as a disease and therefore do not consult a physician [16].

The aim of this study was to describe our experience on allergic rhinitis, associated co-morbidities and treatment outcome among patient attending Bugando Medical Centre. Early identification of patient with allergic rhinitis and its co-morbidities would suggest development of guideline for early detection and proper treatment of allergic rhinitis and so reduce development of co-morbidities.

## Methods

### Study design and setting

This was a descriptive prospective hospital-based study of patients with clinical Allergic rhinitis carried out at Bugando Medical Centre in Northwestern Tanzania over a period of three months from July to September 2011. Bugando Medical Centre is located in Mwanza city along the shore of Lake Victoria in the northwestern part of Tanzania. It is a tertiary care and teaching hospital for the Catholic University of Health and Allied Sciences- Bugando (CUHAS-Bugando) and other paramedics and has a bed capacity of 1000. Bugando Medical Centre is one of the four largest referral hospitals in the country and serves as a referral centre for tertiary specialist care for a catchment population of approximately 13 million people from Mwanza, Mara, Kagera, Shinyanga, Tabora and Kigoma regions.

### Study subjects and procedures

The study included all patients with clinical diagnosis of allergic rhinitis presented with two or more recurrent nasal symptoms of excessive sneezing, watery nasal discharge, nasal congestion and itching of nose and eyes in all age group and sex attended Bugando Medical Centre ENT clinic and ENT wards during the study period. Patients with co-morbid conditions associated with allergic rhinitis like otitis media, nasal polyps, tonsillitis, hypertrophied turbinate and sinusitis were also included in the study. Patient who refused investigations i.e. nasal scrapings and those received intranasal steroid one week prior to the enrollment into the study were excluded from the study. Recruitment of patient to participate in the study was done at ENT clinics and wards. Patients were screened for inclusion criteria and those who met the inclusion criteria were given an explanation about the study and requested to sign a written informed consent for the study before being enrolled in the study. In case of children below 18 years the Guardian/Parents were asked to consent on their behalf. Convenient sampling of patients who met the inclusion criteria was performed until the sample size was reached.

Data were collected using a pre-tested coded questionnaire and physical examination. Data administered in the questionnaire included; patients characteristics (e.g. age, sex, occupation), nasal symptoms, eye symptoms, age of onset of symptoms, triggering factors, presence or absence of family history of allergic rhinitis and asthma in the first and second degree relatives, presence of co morbidity and the effects to the quality of life (defined as interference with daily activities and sleep disturbances). Appropriate examination was done to all patients where candidate with nose complaints were examined using anterior rhinoscopy. All candidates with ear complaints were inspected using otoscope for evidence of eustachian tube dysfunction and otitis media. Children who showed features of allergic rhinitis and history of sleep noisy snoring or apnoea their posterior oropharyngeal wall were examined for adenoid hypertrophy with the aid of wooden tongue spatula. Sinusitis in atopic patient was defined by clinical presentation of facial pain, blocked nose, thicker and purulent rhinorrhoea and or anosmia. Paranasal sinus radiographs X - Ray submental vertical (water) view was taken in all suspected respondents for air/fluid level, septal deviation and thickening mucosa and was interpreted by an experienced radiologist. Extracapsular tonsillectomy and adenoidectomies were done in selected patients who had enlarged tonsils and adenoid hypertrophy which obstruct breathing, causing snoring, and sleep apnoea, difficult in feeding and recurrent bacterial infection.

In selected patients with hypertrophy of inferior turbinate with obstructive nasal symptoms electrical cauterization of submucosa of inferior turbinate under general anaesthesia was done. Patient with chronic maxillary sinusitis were treated by Caldwell-luc procedure.

The research assistant and Principle Investigator gathered relevant information regarding history and physical examination and the Scrapings of secretions and nasal mucosa cells [17] were taken from all patients and eosinophils, neutrophils and basophils count was done. Nasal Eosinophil infiltration is the cornerstone of allergic inflammation [18] and was found to be sensitive for the diagnosis of allergic rhinitis [19,20]. Nasal mucosal specimens were scraped from the surfaces of the middle thirds of inferior turbinate with specially made wooden spatula as rhinoprobes. They transferred onto plain glass slides, fixed in 95% ethyl alcohol for at least 1 minute, excess alcohol was drained. The collected slides were sent to the laboratory for staining. The slides were dipped sequentially in modified Wright- Giemsa stain, for 15 seconds, in Volu-Sol buffer for 30 seconds, and in Volu-Sol hematology rinse for 5 seconds. The slides were drained of excess fluid between the procedures. After air drying, the slides were examined under oil immersion by light microscopy [21,22]. At least 10-well spread, high power epithelium fields were examined. It was known from literature that nasal cytology assist in (1) distinguishing inflammatory from non-inflammatory rhinopathies; (2) distinguishing between allergic, non-allergic, and infectious rhinitis; (3) distinguishing between viral and bacterial infections; (4) following the course of a disease; and (5) following the response to treatment.

The quantitative score of nasal eosinophils and metachromatic cells were rated according to a scale previously described by Meltzer [23]. The grading were semiquantitative on a scale of 0 to 4 + [21]. It was shown that the presence of either nasal eosinophilic or basophilic metachromatic cell provides the best sensitivity and specificity [20,24]. Eosinophil scores and/or basophilic metachromatic cell scores of 1 was considered positive cut-off points for the diagnosis of allergic rhinitis. The diagnosis of patient with allergic rhinitis and associated co-morbidities was made clinically based on the validated questionnaire derived from international studies of allergic rhinitis i.e. Score For Allergic Rhinitis (SFAR) and International Study of Asthma and Allergies in Childhood (ISAAC) questionnaire where they had satisfactory sensitivity and specificity, in the absence of any allergic test in developing countries [3]

All patients with no co morbidities were treated conservatively with intra nasal corticosteroids (Fluticasone) and oral antihistamine citerizine and selected patients with co morbidities were operated accordingly. Patients were followed up until the day of discharge or death. After discharge patients were followed up at our ENT clinic for one month (30 days) period. Length of hospital stay (LOS) and mortality were recorded at the end of study period.

### Statistical data analysis

Data was analyzed using SPSS software version 17.0 with the guide of medical statistician. Data was summarized in the form of proportions frequency tables, bar and pie charts for categorical variables. Appropriate summary was used for continuous variables such as Mean, median, mode, standard deviation and histogram. Chi- square test was used to test for significance of association between predictors and outcome variables in the categorical variable. Odds ratio was calculated to test the strength of association between predictor and outcome variables in the continuous variables. Significance was defined as a p-value of less than 0.05. Multivariate logistic regression analysis was used to determine the predictor variables that are associated with outcome.

### Ethical consideration

Ethical approval to conduct the study was obtained from the CUHAS-Bugando/BMC joint institutional ethic review committee before the commencement of the study. Informed consent was sought from each patient before being enrolled into the study.

## Results

During the study period, a total of 1294 ENT patients were seen at Bugando Medical Centre. Of these, 200 patients had a clinical diagnosis of Allergic rhinitis. Ten patients were excluded from the study due to failure to meet inclusion criteria. Thus, 190 patients were studied representing 14.7% of cases. There were 95 (50.0%) males and 95 (50.0%) females with a male to female ratio of 1:1. The age of the patients raged from 5 months to 56 years with a median age of 8.5 years. The model age group was 0 –10 years accounting 55.8% of cases (Table 1).

**Table 1:** Distribution of patients according to age group

**Age group (Years)**	**Frequency**	**Percentage**
0 – 10	108	56.8
11 – 20	23	12.1
21 – 30	27	14.2
31 -40	15	7.9
41 – 50	11	5.8
51- 60	5	2.6
61+	1	0.5
**Total**	**190**	**100.0**

The majority of patients, 77 (40.5%) were in the pre school age group and occupation of the patients is as shown in the Table 2.

**Table 2:** Distribution of patients according to education and occupation

**Variable**	**Response**	**Frequency**	**Percentage**
**Level of education**	Pre school	77	40.5
	Primary education	44	23.3
	Secondary education	51	26.8
	Tertiary education	18	9.5
	**Total**	**190**	**100**
**Occupation**	Students	68	60.1
	Peasants	18	15.9
	Business	13	11.5
	Teachers	8	7.1
	Mining workers	2	1.8
	Industrial workers	2	1.8
	Health workers	2	1.8
	**Total**	**113**	**100.0**

The age of onset of allergic rhinitis symptoms ranged from 2 months to 56 years with a median age of 3 years. The onset of symptoms were significantly associated with co-morbidities (*χ*^2^ = 10.963, P = 0.001).

The majority of patients, 189 (99.5%) presented with nasal symptoms, of which blocked nose was the most common presentation affecting 75.0% of patients (Table 3). There was statistically significant association between the blocked nose and age below 12 years in the study population. (*χ*^2^ = 9.513, P = 0.002). Ninety-eight (51.6%) patients had eye symptoms (rhinoconjuctivitis), of which watery eye discharge was the most common symptoms affecting 33.2% of cases.

**Table 3:** Distribution of patients according to nasal and eye symptoms

**Symptoms**	**Response**	**Frequency**	**Percentage**
**Nasal symptoms**	Blocked nose	144	75.8
	Runny nose	124	65.3
	Recurrent sneezing	111	58.4
	Nasal itching	101	53.2
**Eye symptoms**	Watery eyes	63	33.2
	Eye itching	62	32.6
**Triggers of the symptoms**	Dust	75	39.5
	Cold weather	53	27.9
	Perfume	34	17.9
	Smoke	7	3.7

Most patients 112 (58.9%) were able to identify the triggers of their allergic symptom and dust was reported to be the most common triggering factor in 39.5% of patients (Table 3). There was a significant association between the dust allergens and sneezing in patient with allergic rhinitis (*χ*^2^ = 12.391, P = 0.002), there was also significant association between sex and strong perfume odors (*χ*^2^ = 4.583, P = 0.032). Seventy-four (38.9%) patients reported to have family history of allergic rhinitis.

In this study, associated sleep disturbance was reported in 141 (74.2%) patients. There was a strong statistical association between sleep disturbance and presence of adenoid hypertrophy (*χ*^2^ = 28.691, P = 0.000) but not with chronic tonsillitis (*χ*^2^ = 2.914, P = 0.088).

One hundred and seventy-seven (93.3%) patients admitted to have interference with their daily activities.

Majority of patients 179 (92.6%) had co-morbidities associated with allergic rhinitis as shown in Table 4. Patients who had allergic rhinitis with co-morbidities had significant interference with quality of life (sleep disturbance and work) compared to allergic rhinitis patients without co-morbidities (*χ*^2^ = 20.711, P = 0.000), and younger age at onset had significant association with development of co-morbidities (*χ*^2^ = 76.040, P = 0.01).

**Table 4:** Distribution of patients according to their co morbidities

**Co morbidities**	**Number of patients**	**Percentage**
Recurrent tonsillitis	105	55.3
Adenoid hypertrophy	88	46.3
Inferior turbinate hypertrophy	77	40.5
Nasal polyps	18	9.5
Ear discharge	16	8.4
Sinusitis	10	5.3

X-ray was taken in 14 patients who had symptoms of sinusitis. Out of these 5 (35.7%) were found to have radiological features of chronic sinusitis. Nasal scrapings were taken in all 190 patients. Neutrophils were found in 177 (77.4%), esinophils 139 (73.2%) and there were no cells found in 18 (9.47%) slides. Eosinophils were higher in females (*χ*^2^ = 7.746, P = 0.005) and in patient with family history of AR (*χ*^2^ = 5.309, P = 0.021).

One hundred and four (54.7%) patients were admitted to hospital for surgical intervention resulting from co morbidities associated with allergic rhinitis (Table 5). Of these, tonsillectomy was the most frequent type of surgical procedure performed accounting for 68.3% of cases. The remaining 86 (45.3%) patients were treated by non-surgical approach (conservatively).

**Table 5:** Distribution of patients according to the type of surgical procedure performed (N = 104)

**Type of procedure**	**Number of patients**	**Percentage**
Tonsillectomy	71	68.3
Adenoidectomy	55	52.9
Polypectomy	7	6.7
Caldwell luc	3	2.9
Turbinectomy	3	2.9

The overall hospital stay for the admitted patients ranged from 2 to 28 days with a mean of 4.6 ± 3.6 days. The median was 3 days.

Postoperative complications were reported in 3 (2.8%) patients who had bleeding post-adenotonsillectomy.

Nine (4.7%) patients were discharged against medical advice. Of these 5 (55.5%) patients were due to long hospital stay before operation. Two (22%) patients were due to financial constrains, and 2 (22%) patient were due to unknown reason.

In this study 3 patients died giving mortality rate of 1.6%. All deaths were attributed to post operative and anesthetic complications. Two (1.9%) patients died from respiratory arrest post operative and one (0.9%) patient died of cardiopulmonary arrest during recovery period.

## Discussion

Allergic rhinitis represents a global healthy problem with considerable prevalence especially in children [25]. The majority of patients in this study were children which is comparable with previous studies done else where [26-29]. The reason for increased number of children with allergic rhinitis to attend medical services in this study may be due to the fact that allergic rhinits is associated with severe and troublesome symptoms which are exacerbated by recurrent viral infections making parents to seek medical attention while majority of symptoms of allergic rhinitis are ignored by adult patients.

In this study, no gender predilection was observed which is in agreement with other studies [30]^,^ but at variant with other studies which reported female [29,31,32] and male [33] predominance respectively.

The prevalence of allergic rhinitis is increasing in developing countries [34] due to the introduction of Western life style [35] and environmental factors [25] and it differs among countries and even among regions within the same country [20]. In industrial countries like USA the allergic rhinitis is one among the well documented disease and high prevalence of allergic rhinitis have been reported to be more than 40% [36]. In the present study the point prevalence of allergic rhinitis at Bugando Medical Centre was 14.7%. This prevalence is comparable to 13.7%,13% found in Kinshasa [37] Nairobi Kenya [30] respectively. Higher prevalence of allergic rhinitis have been reported in African countries such as 29.6% in Nigeria [29] and more than 30% in Cape Town South Africa [11]. Several theories for the increasing prevalence of allergic rhinitis are climatic factors, increasing in winter and rainy seasons, dietary changes, environmental factors including industrial pollution. These factors may also influence the prevalence in our study and therefore it is recommended that future studies should be done in different periods within a year to determine the variations. The prevalence of allergic rhinitis in our study may actually be underestimate as majority of patients with this condition are treated in the peripheral hospitals and only patients with severe symptoms or associated co-morbidities present to ENT surgeon. A better picture of the magnitude of Allergic rhinitis in this region requires comprehensive data collection including both hospital and population-based study.

Clinically allergic rhinitis is defined as allergen induced inflammation of the nasal membrane and surrounding tissues that results in sneezing, rhinorrhoea, conjunctivitis, nasal congestion, and pruritis of the nose, palate, throat and ears [38]. Nasal obstruction is associated with sleep disorders, which can have a profound effect on quality of life, mental health, learning, behavior and attention [39]. Vast majority of respondents in this study had nasal symptoms of which blocked nose was found in many patients and it was found to be the most troublesome symptom. The same observation was also reported in other studies done else where [40,41]. The reason for the nasal obstruction was due to the co-existence with adenoid hypertrophy, nasal congestion, inferior turbinate hypertrophy and nasal polyps in majority of our study population. In this study blocked nose showed significant association with adenoid hypertrophy in children.

In the present study, the high proportion of patients had runny nose (rhinorrhoea) compared to other studies found in literature [42]. The reason for increased proportion of runny nose is the fact that runny nose is common in small children which is commonly associated with viral and bacteria infection especially in under five which were the majority in our study.

The rate of allergic conjunctivitis found in this study is higher as compared to other studies [26,43] and majorities of patients had watery eye discharge as compared to eye itching. Ocular symptoms were considered trivial illness by most of our patients and were not complained. The reason for the high proportion of allergic conjunctivitis could be due to dusty environment which causes symptoms in majorities of our study population. The high prevalence of rhinoconjunctivitis was also found in majorities of the centers of Africa and raises number of questions and non allergic factors are found to be responsible [44]. Further studies are needed to determine the factors responsible for rhinoconjunctivitis.

In the present study it was shown that the allergic rhinitis symptoms could begins as early as one month old child. This is earlier compared to 18 month old reported in Paris [45]. This could be due to differences in environment between the two countries. The study also shows that the early onset of symptoms of allergic rhinitis is significantly associated with the development of co-morbidities. This explains the needs for public awareness and early medical intervention.

Various environmental factors were found to increase the risk of allergic rhinitis especially in children. Pollution factors such as environmental tobacco smoke exposure, moulds, road traffic pollution and dusts seem to be important risk factors of allergic rhinitis [25]. In this study exposure to dust, weather changes, strong perfume order, and smoke were most common self reported triggers for allergic rhinitis where dust reported by majorities of patients. This observation is in agreement with other studies reported elsewhere [2,46].

The proportion of patients with self-reported allergy to dust was 39.5% which is lower than reported in Nigeria [29] and South Africa [11]. Seventy three percent of respondents noted trouble symptoms inside their houses. The reason for these findings could be due to the plenty of dust in our immediate environment and house dust which mainly consists of dust mite, moulds, insects and animal dander may be the etiological trigger in the study population. In this study dust was found to have statistical significant association with sneezing. Sneezing aims to expel mucus containing irritating allergens and cleanse the nasal cavity.

It was found in the present study that 17.9% and 27.9% of patients were affected by strong perfume and cold weather respectively. This rate is lower compared to 31.1% and 32.4% respectively reported in the literature [47]. Female patients with allergic rhinitis showed statistical significant association with strong perfume odor. This might be due to the social habits of using strong perfumes which is commonly in female compared to males.

Many patients with allergic rhinitis had their first degree relatives suffering from the disease. Similar findings were reported in other studies [11,48,49]. Unlike in other studies where family history was significant risk factor for allergic rhinitis [11,45], in the current study family history of allergic rhinitis did not reach statistical significance. The reason for poor association could be due to small sample as compared to the general population. However family history of allergic rhinitis showed statistical significant association with early onset nasal symptoms, which leads to early development of co-morbidities.

The quality of life is often impaired in patients with allergic rhinitis, due to the classic symptoms of the disease (sneezing, pruritis, rhinorrhoea and nasal obstruction). In addition, the pathophysiology of allergic rhinitis often disrupt sleep, leading to fatigue, daytime sleepiness, irritability and memory deficit [39]. Sleep disturbance was reported in most of our study population. Similar observation was also reported from other studies [50]. While 74.2% of our patient had their sleep affected, Machimu *at al*[51] reported sleep disturbance in 76.6% of the study population. Nasal obstruction and nasal congestion were responsible for the sleep disturbances. In this study nasal obstruction and congestion cause day time sleepiness in more than 70% of respondents especially those who had sleep disturbance during night time. This is in agreement with previous study [52]. The reason for the nasal obstruction was adenoid hypertrophy in one hand and hypertrophy of inferior turbinate in another hand. Adenoid hypertrophy was complained in majority of patients and it was significantly associated with sleep disturbance in children. Another cause of nasal obstruction was rhinorrhoea triggered by allergens.

Most of our patients 93.3% reported to have interference with their daily activities such as playing, working etc. and thus they have impaired social life. This is in agreement with other studies found in literature [53,54] and it is higher than that reported by Bousquet [55]. The reason for the interference with daily activities is probably due to severe symptoms of nasal blockage and rhinorrhoea which also causes embarrassment to patients.

Besides its direct effect on the quality of life, allergic rhinitis has significant co-morbid disorders such as asthma, sinusitis, otitis media, conjunctivitis and adenoid hypertrophy [25]. In the present study, more than 90% of patients had associated co-morbidities which is contrary with other studies [34]. Many other studies reported low incidence of associated co-morbidities. We could not find the reason for these differences, this call for further studies to explain these differences.

Multidisciplinary approach is needed in the treatment of allergic rhinitis and its co-morbidities involving paediatricians, allergists and the otolaryngologists [25]. Allergen avoidance require aggressive environmental control which is effective but often practically difficult [56]. Intranasal steroids are the treatment of choice and are more effective than antihistamines for relief of nasal obstruction however surgical therapy is reserved for co-morbidities refractory to medical treatment. Immunotherapy may also be used [57].

With regard to treatment patterns, majority of patients (54.7%) in this study underwent surgical treatment which is at variant with findings from other studies [11] where majority of allergic rhinitis patient were treated conservatively and surgical management was reserved for minority of cases who were refractory to medical treatment. The most common indications for surgical treatment in the present study were adenoid hypertrophy, recurrent tonsillitis, nasal obstruction due to hypertrophy of inferior turbinate, nasal polyps and sinusitis. The high rate of surgical treatment found in this study is attributed to high proportion of patients with associated co-morbidities requiring surgical interventions

In agreement with previous report [58], conservative treatment in this study was done using mono therapy with steroid nasal spray fluticasone which does not result in complete relief of symptoms and so prolongs the hospital visit and affect compliance with long term use.

Allergen exposure in atopic individual activates mast cells resulting in the release of mediators and cytokines capable of inducing inflammatory cell recruitment including eosinophils, neutrophils and basophils at the target organ level. Eosinophils in the nasal smear has shown to display the best correlation with clinical allergic rhinitis [59], and can be used not only to establishes the diagnosis of allergic rhinitis but also useful in the follow up of patients with this condition [22]. In this study oesinophils were found in 73.2% of patients and were significantly associated with female sex and family history of allergic rhinitis. The reason for this association was not known

Polymorpho-nuclear cells were found in 77.4% of our patients. Since the presence of neutrophils provides evidence substantiating the diagnosis of nasal infections, high rate of nasal neutrophils in this study indicate that nasal infections is common in these groups.

Nasal smears for oesinophils, basophils and neutrophils were negative in 9.7% of the slides. Unlike observation from other studies that nasal mucosa scrapings in allergic rhinitis patients had large numbers of basophilic metachromatic cells [60] in this studies no basophils was found. The reason could be due to the fact that basophil cells are found predominately in the nasal mucosa [23], where proper nasal curettes (rhino-probe) are required for acquisition of a large number of intact cells to interpret. In contrary neutrophils and oesinophils are present in both nasal secretion and within the nasal mucosa.

The overall average length of hospital stay for in patients was 4.6 days. However for the patient underwent adenotonsillectomy the average hospital stay was 3 days. This was in contrary with other studies done in Malaysia where adenotonsillectomy was done safely as day care surgery [46].

While gender was not found to be a risk factor, younger age group was significantly associated with increased length of hospital stay post adenotonsillectomy. Same observation was also reported in the literature [61]. This is contrary with other studies [62]. The reason for increased length of hospital stay was mostly due to delay in conduction of the procedure, where patients stayed in the ward for days awaiting surgery.

Patients who had chronic sinusitis were found to have statistical significant association with increased length of hospital stay. The reason for this is that during post operative period, most of patients had associated oedema and prolonged catheter placed for sinus drainage.

In the present study post operative bleeding was the most post surgical complication. This is consistent with other study [63]. The rate of post operative bleeding of 2.88% in the present study was in agreement to the rate of 2.4% found in other studies [64]. The low rate of post tonsillectomy bleeding was attributed to the suture tie techniques used to arrest bleeding during tonsillectomy at Bugando Medical Centre and also weekly follow up post surgery was found to be effective in early detection of delayed and episodic bleeding which can be corrected before the development of life-threatening anaemia.

Majority of patients were discharged against medical advice in the current study was caused by long hospital stay especially before surgery. This is attributed to inadequate operating days for ENT patients despite of high number of patients demanding surgical services. Further more lack of essential surgical equipments such as oxygen, gauze and surgical gowns during the study period led to delay of surgical procedures.

AR is not common to cause death but it may occur from its co-morbid conditions as 1.5% mortality rate reported in a study done in Sweden [65] or as a result of surgical complications. In the current study death rate post adenotonsillectomy was 1.6%. High mortality rate was previously reported [66] although there was no postoperative deaths reported in study done in Germany [67]. In our study death occurred within the first 24 hours post adenotonsillectomy in younger children who had severe obstructive symptoms. Aspiration and respiratory arrest may probably be the cause of death. This calls for improvement of pediatric post operative care.

There was no death caused by bleeding in this study. The reason was careful inspection of the nasopharynx before performing surgery and curettage in a piecemeal fashion under visual control which was done during each adenoidectomy in order to prevent direct injury to aberrant arteries.

The potential limitations of this study include the absence of allergy testing, short study period, presence of neutrophils in the nose and failure of estimating the measure of quality of life based on a standardized and validated (generic or disease-related) questionnaires. However, despite these limitations, the study has provided local data that can help health care workers develop guideline for early detection and proper treatment of allergic rhinitis and so reduce development of co-morbidities.

## Conclusion

Allergic rhinitis is common condition which is under diagnosed in patients attending otorhinolaryngology department at Bugando Medical Centre and accounts for 14.7% of all otorhinolaryngology admissions. Allergic rhinitis causes considerable morbidities especially in children. Many of the allergic rhinitis triggers are commonly found in our environment and houses. It is therefore recommended that:

High index of suspicion and improvement of laboratory diagnostic facilities for allergic rhinits are needed in order elevate the prevalence of this disease.

Early detection and treatment of allergic rhinitis in children is needed to improve healthy of the sufferers

Avoidance of allergic rhinitis trigger is the best first line of management of allergic rhinits and so all patients should be given education on avoidance of the triggering factors.

Further study on incidence and risk factors of allergic rhinitis at Bugando Medical Centre ENT patients is highly recommended.

## Competing interests

The author declares that they have no competing interest.

## Authors' contributions

SAS designed the study, contributed in literature search, data collection and management of patients. JMG participated in study design, treatment of patient and performed surgery. PLC designed the study, data analysis, editing, writing and submission of the manuscript. PR participated in editing and preparation of nasal smears and microscopic interpretations. MDM participated in data analysis, supervising the study. All the authors read and approved the final manuscript.

## Pre-publication history

The pre-publication history for this paper can be accessed here:

http://www.biomedcentral.com/1472-6815/12/13/prepub
